# Zika virus infection in Malaysia: an epidemiological, clinical and virological analysis

**DOI:** 10.1186/s12879-019-3786-9

**Published:** 2019-02-13

**Authors:** Yuan Liang Woon, Mei Fong Lim, Tg Rogayah Tg Abd Rashid, Ravindran Thayan, Suresh Kumar Chidambaram, Syed Sharizman Syed Abdul Rahim, Rose Nani Mudin, Sheamini Sivasampu

**Affiliations:** 1 Clinical Epidemiology Unit, National Clinical Research Centre, Ministry of Health Malaysia, Level 2, Block B4, National Institute of Health, Jalan Setia Murni U13/52, Seksyen U13, 40170 Shah Alam, Selangor Darul Ehsan Malaysia; 2 Healthcare Statistics Unit, National Clinical Research Centre, Ministry of Health Malaysia, Level 4, Block B4, National Institute of Health, Jalan Setia Murni U13/52, Seksyen U13, 40170 Shah Alam, Selangor Darul Ehsan Malaysia; 3 Virology Unit, Infectious Disease Research Centre, Institute for Medical Research, Jalan Pahang, 50588 Kuala Lumpur, Malaysia; 4 Department of General Medicine, Hospital Sungai Buloh, Jalan Hospital, 47000 Sungai Buloh, Selangor Darul Ehsan Malaysia; 5 Sector of Vector-Borne Disease, Disease Control Division, Ministry of Health Malaysia, 62590 Putrajaya, Malaysia

**Keywords:** Zika, Malaysia, Epidemiology

## Abstract

**Background:**

A major outbreak of the Zika virus (ZIKV) has been reported in Brazil in 2015. Since then, it spread further to other countries in the Americas and resulted in declaration of the Public Health Emergency of International Concern (PHEIC) by World Health Organization. In 2016, Singapore reported its first minor ZIKV epidemic. Malaysia shares similar ecological environment as Brazil and Singapore which may also favor ZIKV transmission. However, no ZIKV outbreak has been reported in Malaysia to date. This study aimed to discuss all confirmed ZIKV cases captured under Malaysia ZIKV surveillance system after declaration of the PHEIC; and explore why Malaysia did not suffer a similar ZIKV outbreak as the other two countries.

**Methods:**

This was an observational study reviewing all confirmed ZIKV cases detected in Malaysia through the ZIKV clinical surveillance and Flavivirus laboratory surveillance between June 2015 and December 2017. All basic demographic characteristics, co-morbidities, clinical, laboratory and outcome data of the confirmed ZIKV cases were collected from the source documents.

**Results:**

Only eight out of 4043 cases tested positive for ZIKV infection during that period. The median age of infected patients was 48.6 years and majority was Chinese. Two of the subjects were pregnant. The median interval between the onset of disease and the first detection of ZIKV Ribonucleic Acid (RNA) in body fluid was 3 days. Six cases had ZIKV RNA detected in both serum and urine samples. Phylogenetic analysis suggests that isolates from the 7 cases of ZIKV infection came from two clusters, both of which were local circulating strains.

**Conclusion:**

Despite similar ecological background characteristics, Malaysia was not as affected by the recent ZIKV outbreak compared to Brazil and Singapore. This could be related to pre-existing immunity against ZIKV in this population, which developed after the first introduction of the ZIKV in Malaysia decades ago. A serosurvey to determine the seroprevalence of ZIKV in Malaysia was carried out in 2017. The differences in circulating ZIKV strains could be another reason as to why Malaysia seemed to be protected from an outbreak.

## Background

Zika virus (ZIKV) is an emerging mosquitoes-borne flavivirus which was first discovered in the Zika forest of Uganda in 1947 [1]. Since then, only intermittent human cases were reported in Asia and Africa; until 2007, when a major epidemic occurred on Yap Island in the Federated States of Micronesia [2, 3]. The infections then shift eastward to French Polynesia and other Pacific Islands in 2013–2014 [1, 4], reached Brazil in 2015 [5, 6], and dispersed further to other countries in South America [7]. As of 2016, ZIKV has spread much further to the North Americas and Asia [1, 7, 8].

On 1st February 2016, World Health Organization (WHO) declared a Public Health Emergency of International Concern (PHEIC) over major worries regarding an association between the ZIKV disease and microcephaly and other neurological disorders in the epidemic region of Latin America and the Pacific Islands [9]. Subsequently, the Ministry of Health (MoH) Malaysia introduced precautionary measures against ZIKV, which includes establishing clinical and laboratory surveillance of Zika infection as well as enhancing vector control activities and crisis response towards imported cases.

In Malaysia, the ZIKV has been isolated in *Aedes aegypti* mosquitoes in 1966 [10]. It is the same vector which transmits other flaviviruses such as dengue, Japanese Encephalitis and Yellow Fever. Dengue virus and ZIKV are antigenically related flaviviruses that elicit similar T-cell responses and antibodies that could cross react with each other [11–14]. Some studies suggested that previous dengue infection may provide protection against ZIKV [15, 16]. Having said that, Brazil with a background dengue seroprevalence ranging from 48.4 to 97.8% among adults aged 18–65 years [17], was badly affected by ZIKV infection in 2015 [5, 6]. Singapore, another dengue endemic country also reported a minor ZIKV epidemic in 2016 although its magnitude was much smaller compared to the outbreak in Brazil [18, 19]. Similar to as Brazil and Singapore, Malaysia is hyperendemic with dengue [20] and has favourable ecological conditions for transmission of ZIKV. However, no ZIKV outbreaks have been reported in Malaysia so far.

Till date, there are no local comprehensive reports on the systematic surveillance of ZIKV are available. Surveillance efforts for ZIKV were increased in Malaysia from September 2016 after the declaration of PHEIC by the WHO in February 2016. This study aims to report epidemiological, virological and clinical findings related to the Zika infection cases detected between June 2015 and December 2017. This study also intends to discuss the situation of ZIKV transmission observed in Malaysia compared to other dengue-endemic countries such as Brazil and Singapore.

## Methods

### Study setting and populations

This was an observational study, which reviewed all confirmed ZIKV cases detected in Malaysia through ZIKV clinical surveillance and Flavivirus laboratory surveillance between June 2015 and December 2017. In the ZIKV clinical surveillance, clinically suspected ZIKV cases would be notified and had their blood and/or urine specimens sent to nearby listed public or private laboratory for real-time reverse transcription polymerase chain reaction (rRT-PCR). The presence of ZIKV antibodies was tested using the following commercial kits: Anti-Zika Virus IgG ELISA (Euroimmun, Germany) and ZIKV Detect™ IgM Capture ELISA (InBios, Seattle, WA, USA). All ZIKV IgM / IgG positive cases were also tested for dengue cross-reactivity using Anti-Dengue Virus IgG ELISA (Euroimmun, Germany) and DENV Detect™ IgM Capture ELISA (InBios, Seattle, WA, USA).

The Flavivirus laboratory surveillance was established since December 2015 [21]. In this surveillance, five cases will be sampled randomly each week from each sentinel site and had their serum specimens sent to the National Public Health Laboratory (NPHL) to test for Flaviviruses (dengue, Japanese encephalitis, West Nile Virus, Yellow fever, ZIKV, Bagaza virus and Usutu virus) and Chikungunya virus. The sampled cases must present to the sentinel sites within 5 days of illness onset, with signs and symptoms similar to dengue and tested negative for dengue non-structural protein-1 (NS1).

The list of confirmed ZIKV cases was obtained from the Vector Borne Disease Control Division, MoH Malaysia. All basic demographics, co-morbidities, clinical, laboratory and outcome data of the confirmed ZIKV cases were collected from the source documents, which included the medical records as well as investigational and laboratory reports. The Medical and Research Ethics Committee (MREC) from Ministry of Health (MOH) approved the study (NMRR-16-1718-32,614).

### Case definition

Suspected case of ZIKV infection is defined as patient who had recent history of travelling to country or geographical area with known local ZIKV transmission (within 1 week prior to disease onset) or residing in the affected area or history of contact with a confirmed ZIKV case; and presented with rash (usually pruritic and maculopapular) and at least two or more of the following: fever, arthralgia, arthritis / periarticular oedema, or conjunctivitis (non-purulent/hyperemic) [22]. This definition was adapted from the case definition used by the Pan American Health Organization (PAHO) and WHO regional office for the Americas [23]. On the other hand, a confirmed case of ZIKV infection is defined as patient who meets both the criteria for a suspected case and has ZIKV ribonucleic acid (RNA) detected in blood and/or urine samples through rRT-PCR [22].

### Phylogenetic analysis

The rRT-PCR method was adapted from Lanciotti et al. (2007) with some modifications [3]. Strains from all positive cases were then sequenced using Sanger sequencing for genomic material characterization. The chromatograms were analysed using ChromPro (Version 2.0) software. The alignment was carried out using Molecular Evolutionary Genetics Analysis (MEGA version 5.05) while phylogenetic tree was generated using the Clustal X software. The sequences were searched using Basic Local Alignment Search Tool (BLAST) and deposited in the GenBank with accession numbers KX906953.1, KX906955.1, KX906954.1, MH130043.1, MH130044.1, KX906956.1 and MH130042.1.

### Statistical analysis

Data were compiled and analyzed using Microsoft Excel 2017. All data were anonymized during analysis. Descriptive analysis was done to describe background characteristics, clinical presentations, laboratory findings and clinical outcome of the included cases. All categorical variables were presented as frequencies and percentages; while continuous variables were presented in median and interquartile range.

## Results

### Demographic profiles

A total of 250 cases fulfilled the case definition and were notified and screened for ZIKV infection through the ZIKV clinical surveillance [24]. After laboratory testing, only seven cases were confirmed as ZIKV infection. Also, 3793 cases were tested for ZIKV infection through the Flavivirus laboratory surveillance and only one case was detected positive for ZIKV. For the remaining cases, 2139 (56.4%) tested positive for dengue, 1517 (40%) were negative for Flaviruses and 136 (3.6%) tested positive for Chikungunya. These eight confirmed ZIKV cases detected through both surveillance programs were included for further discussion in this study (Table 1). As part of public health efforts to control ZIKV transmission, active case detection was conducted subsequently within 400 m from the index cases. Total of 2767 cases were screened in active detection but all samples tested negative.

**Table 1:** Demographic Profile of Confirmed ZIKV Cases in Malaysia

Demographic characteristics	
Age (years)	
Median (Interquartile range)	48.6 (31.5, 60.8)
Minimum	25
Maximum	61
Sex, n (%)	
Male	3 (37.5)
Female	5 (62.5)
Ethnicity, n (%)	
Malay	1 (13.0)
Chinese	5 (62.5)
Indian	0 (0.0)
Others	2 (25.0)
Comorbidity, n (%)	
Coronary heart disease	3 (37.5)
Hypertension	2 (25.0)
Dyslipidaemia	2 (25.0)
Diabetes mellitus	1 (13.0)
Obesity	2 (25.0)
Chronic kidney disease	1 (13.0)
History of dengue infection	1 (13.0)
Pregnant, n (%)	2 (25.0)

Patients’ ages ranged between 25 and 61 years, with a median of 48.6 years. Most of the patients were Chinese. All patients were discharged well from health care facilities except a male patient who succumbed to acute myocardial infarction with myocarditis. The patient also had underlying Zika and dengue virus co-infection, in which the dengue infection was confirmed by Multiplex dengue real time RT-PCR. Out of five females with confirmed ZIKV infection, two of them were pregnant at 10 and 17 weeks of gestation respectively. One of the pregnant women was lost to follow-up while the other delivered a baby at term without congenital Zika syndrome. At birth, the newborn’s serum and urine tested negative for ZIKV based on rRT-PCR. During a 2-year follow-up period, clinical and developmental assessments revealed normal development. Findings from ophthalmology examination by fundoscopy, hearing assessment by auditory brainstem response and neuro-imaging by cranial ultrasound were unremarkable.

### Clinical presentation

Rash was the most common clinical manifestation, which was observed in nearly all patients, followed by fever and myalgia (Table 2). None of the patients had headache upon presentation and only one patient complained of conjunctivitis.

**Table 2:** Clinical Presentation of Confirmed ZIKV Cases in Malaysia

Clinical Presentation	Frequency, n (%)
Rash	7 (87.5)
Fever	6 (75.0)
Myalgia	3 (37.5)
Arthralgia	2 (25.0)
Cough	2 (25.0)
Conjunctivitis	1 (12.5)
Retro-orbital pain	1 (12.5)
Headache	0 (0.0)

### Laboratory findings

Eight cases were confirmed with ZIKV infection based on the detection of ZIKV genome in body fluids by rRT-PCR. The median duration between onset of disease and first detection of ZIKV genome in body fluids was 3 days (IQR: 3, 4.5). Six out of eight cases had ZIKV RNA detected in both serum and urine samples; but other two patients had ZIKV RNA detected in either serum or urine samples only (Table 3). In addition, five of the confirmed ZIKV cases also had evidence of ZIKV immunoglobulin M (IgM), indicating recent infection. Median time to detection of ZIKV IgM was 5 days (IQR: 4, 6). Although ZIKV IgG was detected in the five cases, there was also evidence of cross reactivity between Zika and Dengue IgG antibodies [13, 25, 26]. Median time to detection of ZIKV IgG was 4 days (IQR: 4, 5.5).

**Table 3:** Summary of Laboratory Findings of Confirmed ZIKV Cases

Patient	Zika rRT-PCR		Zika IgM	Zika IgG	DENV IgM	DENV IgG
	Sample Type	Result				
Patient 1	Serum^a^	Detected	–	–	–	–
	Urine^a^	Detected				
Patient 2	Serum	Not Detected	Reactive	Non-reactive	Non-reactive	Non-reactive
	Urine	Detected				
Patient 3	Serum	Detected	Non-reactive	Reactive	Non-reactive	Reactive
	Urine	Detected				
Patient 4	Serum	Detected	Reactive	Reactive	Non-reactive	Reactive
	Urine	Detected				
Patient 5	Serum	Detected	Reactive	Reactive	Non-reactive	Reactive
	Urine	Detected				
Patient 6	Serum	Detected	Reactive	Reactive	Non-reactive	Reactive
	Urine	Not Detected				
Patient 7	Serum	Detected	Reactive	Reactive	Non-reactive	Reactive
	Urine	Detected				
Patient 8	Serum	Detected	Non-reactive	Non-reactive	Non-reactive	Reactive
	Urine	Detected				

^a^insufficient sample to proceed with serological tests

*rRT-PCR* real time Reverse Transcription Polymerase Chain Reaction, *DENV* Dengue virus, *Ig* Immunoglobulin

**Fig. 1:**
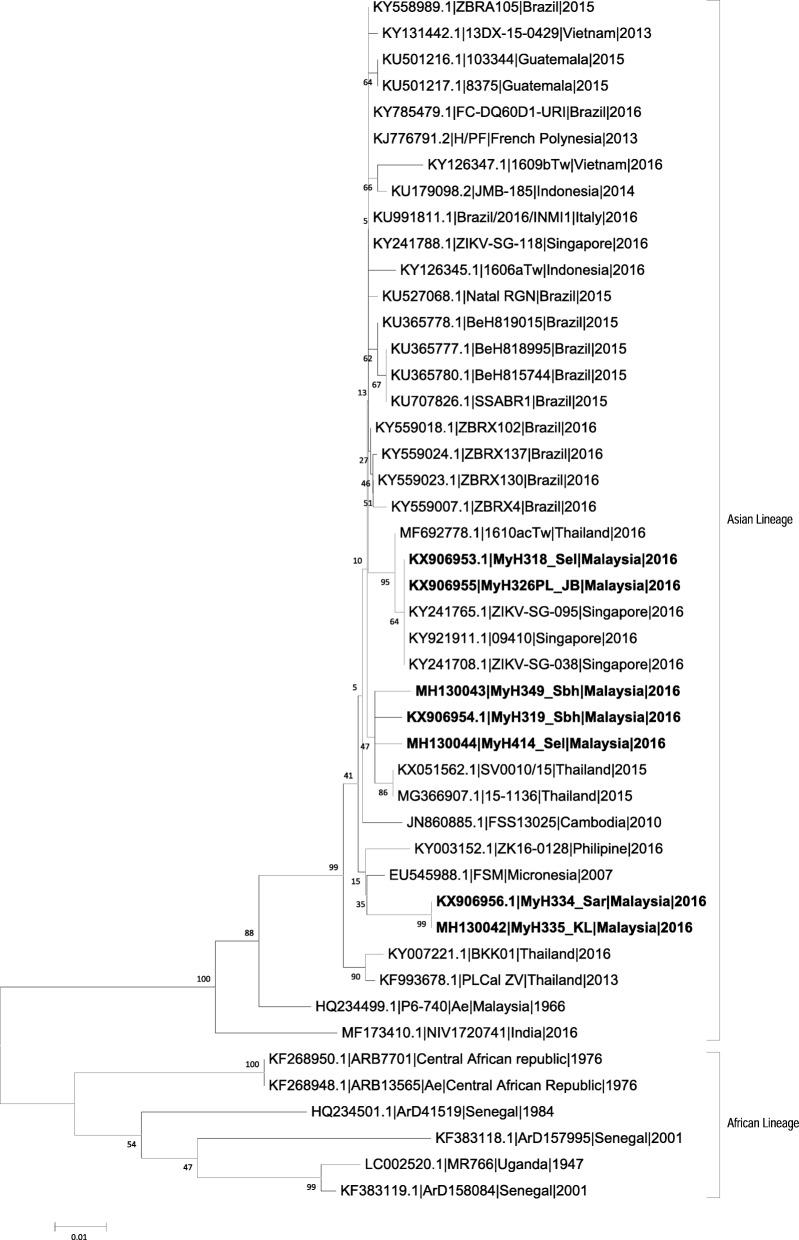
Phylogenetic tree of partial envelope gene (574 bp) of the Zika virus. The tree was inferred using the Neighbor-Joining method [62]. The tree was drawn to scale, with branch lengths in the same units as those of the evolutionary distances used to infer the phylogenetic tree. The evolutionary distances were computed using the Maximum Composite Likelihood method [63] and were in the units of the number of base substitutions per site. The analysis involved 36 nucleotide sequences. Evolutionary analyses were conducted in MEGA5.05 [64]

In terms of phylogenetic analysis, only a 574 base pair (bp) fragment of the envelope gene of the virus were successfully sequenced due to low copy number of the virus from the study subjects. The results indicate that there are two clusters of ZIKV strains where two strains (MyH318 and MyH326) had clustered with strains from Singapore (2016) while the remaining five strains (MyH319, MyH349, MyH414, MyH334 and MyH335) were clustered into another group which includes older ZIKV strains such as the Micronesian (2007) and Cambodian (2010) strains (Fig. 1).

## Discussion

### Clinical features & laboratory findings

Generally, the ZIKV infection can produce diverse clinical symptoms in humans, ranging from asymptomatic presentation to flu-like symptoms [1, 27, 28]. The common symptoms associated with the ZIKV infection include fever, rash, non-purulent conjunctivitis, arthritis and arthralgia [29, 30]. This is reflected in our study where most of the patients complained of fever upon presentation. As rash was one of the mandatory criteria for ZIKV screening in clinical surveillance, all cases included in this study complained of rashes except one which was detected through the Flavivirus laboratory surveillance.

As compared to other modes of detection, rRT-PCR is the most common method used in diagnosing ZIKV infection because of its specificity and ability in differentiating ZIKV from other flavivirus infections [31]. However, rRT-PCR on serum sample is found to have lower sensitivity owing to low and short duration of viremia in humans [32]. Previous literature suggested detection of higher viral RNA load in urine samples for longer duration [33, 34], therefore it is recommended to perform rRT-PCR on both blood and urine samples in order to increase test sensitivity, particularly during the late stage of infection [35]. In this study, both serum and urine samples were tested for ZIKV genome. The detection rate of ZIKV RNA in both serum and urine samples were similar.

### Phylogenetic analysis

Phylogenetic analysis was conducted to determine genetic relationship among all ZIKV strains detected in 2016 as well as with the 1966 ZIKV strain from Bentong, Malaysia. Interestingly, two Malaysian strains (MyH318 and MyH326) had clustered with strains from Singapore (2016) and were from patients with history of travel to Singapore. On the other hand, all the remaining five Malaysian strains (MyH319, MyH349, MyH414, MyH334 and MyH335) were clustered with the Micronesian (2007) strain, and came from patients with no history of recent travel. This indicated that they were all infected with locally circulating ZIKV strains. These findings are important as it provides evidence that ZIKV is circulating amongst our population and there are possible risks to pregnant women and their babies.

### Public health measures taken to detect and contain ZIKV transmission

The detection rate of ZIKV infection among cases which were tested for ZIKV infection was 0.2%. The index cases were sporadic and no ZIKV outbreak was observed during the study period. This observation could be attributed to measures taken by MoH Malaysia after declaration of PHEIC. According to the standard operating procedure [22], once a confirmed Zika case was reported, the district health office has to conduct active case detection towards household members and close contacts of the index cases, as well as any households within a 400 m radius from the index case that have any signs and symptoms of ZIKV infection. All cases would have their blood and urine samples taken for confirmation of ZIKV infection.

During active case detection, the Malaysian Public Health Division also incorporated a series of control and preventive strategies to contain ZIKV transmission from the index cases. These include source reduction, larviciding, fogging and ultra-low volume (ULV) spray within a 400 m radius from the index case. These measures were taken within 24 h after the notification of index cases, which could potentially contain the ZIKV transmission from index cases.

### Comparison of ZIKV infection in Malaysia with Brazil and Singapore

In 2015, Brazil reported its first autochthonous transmission of ZIKV [5, 6]. It caused an epidemic of ZIKV in at least 14 Brazilian states, with an estimated 440,000 to 1,300,000 suspected ZIKV cases being reported [7]. Since then, ZIKV has spread at an alarming rate throughout Central and South America and the Caribbean [1, 7, 8]. Following this outbreak, Malaysia has also reported its first autochthonous Zika case in September 2016.

ZIKV infection in Malaysia was not as widespread compared to the outbreaks in neighbouring countries such as Singapore and Thailand [36]. The incidence of ZIKV infection in Singapore and Thailand was about 0.81 and 0.16 per 10,000 population respectively in year 2016 [18, 37], as compared to Malaysia where the incidence was less than 0.01 per 10,000 population. Overall, there were only eight clinical cases of Zika as most cases of Zika would have been missed due to the asymptomatic nature of the infection. Unlike the dengue virus with four different serotypes, previous infection of ZIKV provides protection against re-infection of ZIKV [38]. Hence, among possible reasons, it could be the higher seroprevalence of Zika in Malaysia that makes clinical cases of Zika uncommon. This assumption is based on the history of Zika in Peninsular Malaysia, which started in the 1960s when the virus was isolated *from Aedes aegypti mosquitoes* in Bentong, Pahang [10]. Another ZIKV seroprevalence study by Wolfe ND et al. showed that 44.1% of the local study population in 1996–1997 were sero-positive for ZIKV [39]. Subsequently, the only other clinical case of Zika was reported in a German tourist who visited Sabah in 2014 [40]. Based on this evidence, it is highly likely that ZIKV has been circulating in the Malaysian population for decades. Because of this, the community would have been provided a respite from large Zika outbreaks. However, whether this also translates into protection against fetal abnormalities needs further research.

Although our phylogenetic analysis showed that the cases of ZIKV detected in Malaysia during 2016 originated from two different sources, it appears that all the isolates were closely related to the old ZIKV strains that have been circulating in South East Asia. On the other hand, studies have shown that the South American ZIKV isolates formed a unique clade within the Asian lineage, which was also demonstrated in our study [41, 42]. Current literature suggested that the American strain of ZIKV seemed to transmit the virus more efficiently through *Aedes aegypti* [43], and resulted in more serious brain damage in mice as compared to other old Asian strain of ZIKV [44]. This difference of the circulating ZIKV strains could be another explanation as to why Malaysia was not as badly affected by the recent ZIKV outbreak in Brazil.

On the other hand, questions arise to why ZIKV transmission in Malaysia does not seem to be as pervasive as in the neighbouring nation, Singapore although both countries are similar in terms of its ecological environment and circulating ZIKV strains [45]. Singaporean authorities reported a total of 455 cases of ZIKV infection within 3 months in 2016 [18] compared to the eight cases in Malaysia in the same year. The explanation for this observation remains unclear, but one theory is that Singaporean population may have a lower prevalence of immunity towards ZIKV infection than Malaysia. Also, transmission of ZIKV is partially dependent on its vector population. Comparatively, Singapore has a smaller *Aedes* mosquito population [46–49]; which may result in a low transmission setting and subsequently give rise to population with low background immunity against ZIKV.

In addition, being a city-state with very small land size compared to Malaysia, Singapore is able to conduct more extensive active case detection, contact tracing, disease surveillance and education of public and health care professionals; which may have contributed to the higher pick up rate of sub-clinical ZIKV cases. Another postulation for the difference in case detection between Malaysia and Singapore could be attributed to the low perception of disease severity among Malaysian. About half of Malaysians surveyed do not consider that Malaysia at risk of ZIKV transmission [50], and a study proved that community-based mosquito control practices did not differ before and after declaration of PHEIC by WHO [51].

### Strengths and limitations

This study discussed all confirmed ZIKV cases captured under the Malaysian ZIKV clinical surveillance and Flavivirus laboratory surveillance. All cases notified to the Malaysian ZIKV clinical surveillance system had both blood and urine samples tested with rRT-PCR for confirmation of ZIKV infection. Although the duration of detectable ZIKV RNA in serum is relatively short, studies had shown ZIKV RNA remain detectable in urine for a longer duration after becoming undetectable in the serum [33, 35, 52, 53]. Because patients sought medical attention while they still had symptoms, it is unlikely that screening of both serum and urine samples would miss out on positive cases of ZIKV infection. In addition, this study also explored why Malaysia did not experience an outbreak to the scale of those observed in Brazil and Singapore although all three countries are dengue-endemic countries and share similar ecological environments.

However, this study is limited by a few limitations. Firstly, the rRT-PCR assay used for the detection of ZIKV infection was adapted from Lanciotti et al. (2007), which its primer sets were designed based on the sequence of ZIKV population in Africa and South America [3]. Therefore, there is concern with its ability to amplify the ZIKV population in Southeast Asia (SEA). However, previous validation studies had reported the high analytical sensitivity of this assay in detecting both African and Asian lineage [54, 55]. Secondly, we were unable to sequence the whole viral genome, hence the partial E region rather than the whole genome was used for phylogenetic analysis. This could possibly result in discrepancy between our phylogenetic tree compared to one which uses the whole genome. However, many published studies also used the partial E region for phylogenetic analysis of the ZIKV [42, 56, 57]. Thirdly, we acknowledge the possible misdiagnosis or under-detection of ZIKV infection for patients who did not fulfill the screening criteria. In this study, we adapted the case definition used by PAHO and WHO regional office for the Americas, in which rash is one of the mandatory criteria for ZIKV screening. Although rash is one of the most common symptoms among ZIKV patients, published literature reported its prevalence to vary between 77 and 100% [2, 18, 27, 58–60]. Therefore, we could potentially miss out some cases which did not present with rash. Lastly, the ZIKV clinical surveillance system in Malaysia is mainly passive where its reporting system is solely dependent on notification by health care providers. Active ZIKV case detection only takes place during the process of contact tracing after an index case has been identified. Hence, there is a possibility of under-reporting as majority of cases were sub-clinical [1], and patients may not seek for medical attention if it was a mild disease.

## Conclusion

This study summarized all ZIKV cases detected in Malaysia after declaration of PHEIC by WHO. Despite similar background characteristics, Malaysia was not affected by ZIKV as much as Brazil and Singapore. This could be related to the development of immunity against ZIKV in the population due to the introduction of ZIKV in Malaysia decades ago. This hypothesis will soon be answered by a study conducted by MOH Malaysia which investigated ZIKV seroprevalence in Malaysia. The local circulating ZIKV strains is different from the South American cluster, which could possibly explain why Malaysia seemed to be protected from a ZIKV outbreak.

## Abbreviations

BLAST: Basic Local Alignment Search Tool
DENV: Dengue virus
IgG: Immunoglobulin G
IgM: Immunoglobulin M
MOH: Ministry of Health
MREC: Medical Research Ethics Committee
NMRR: National Medical Research Register
PAHO: Pan American Health Organization
PHEIC: Public Health Emergency of International Concern
RNA: Ribonucleic Acid
rRT-PCR: Real-time Reverse Transcription Polymerase Chain Reaction
SEA: Southeast Asia
WHO: World Health Organization
ZIKV: Zika Virus
